# Towards achieving the 90–90–90 HIV targets: results from the south African 2017 national HIV survey

**DOI:** 10.1186/s12889-020-09457-z

**Published:** 2020-09-09

**Authors:** Edmore Marinda, Leickness Simbayi, Khangelani Zuma, Nompumelelo Zungu, Sizulu Moyo, Lwando Kondlo, Sean Jooste, Patrick Nadol, Ehimario Igumbor, Cheryl Dietrich, Melissa Briggs-Hagen

**Affiliations:** 1 Human Sciences Research Council, Pretoria, South Africa; 2 School of Public Health, University of the Witwatersrand, Johannesburg, South Africa; 3 Department of Psychiatry and Mental Health, University of Cape Town, Cape Town, South Africa; 4 School of Public Health, University of Cape Town, Cape Town, South Africa; 5 Division of Global HIV and TB, Centers for Disease Control and Prevention, Atlanta, Pretoria, GA South Africa; 6 School of Public Health, University of the Western Cape, Cape Town, South Africa

**Keywords:** 90–90–90 indicator, Diagnosed, ART, Viral suppression, South Africa

## Abstract

**Background:**

Measuring progress towards the Joint United Nations Programme on HIV/AIDS (UNAIDS) 90–90–90 treatment targets is key to assessing progress towards turning the HIV epidemic tide. In 2017, the UNAIDS model estimated that 75% of people living with HIV (PLHIV) globally knew their HIV positive status, 79% of those who knew their status were on antiretroviral therapy (ART), and 81% of those who knew their HIV status and were on ART had a suppressed viral load. The fifth South African national HIV sero-behavioural survey collected nationally representative data that enabled the empirical estimation of these 90–90–90 targets for the country stratified by a variety of key factors.

**Methods:**

To evaluate progress towards achievement of the 90–90–90 targets for South Africa, data obtained from a national, representative, cross-sectional population-based multi-stage stratified cluster random survey conducted in 2017 were analysed. The Fifth South African National HIV Prevalence, Incidence, Behaviour and Communication Survey (SABSSM V), collected behavioural and biomarker data from individuals residing in households from 1000 randomly selected Small Area Layers (SALs), across all nine provinces of the country. Structured questionnaires were used to collect socio-demographic data, knowledge and perceptions about HIV, and related risk behaviours. Blood samples were collected to test for HIV infection, antiretroviral use, and viral suppression (defined as < 1000 copies/ml). Weighted proportions of study participants aged 15 years and older who tested HIV positive were computed for those who reported awareness of their status (1st 90), and among these, those who were currently on ART (2nd 90) and of these, those who were virally suppressed (3rd 90).

**Results:**

Among persons 15 years and older who were HIV positive, 84.8% were aware of their HIV positive status, of whom 70.7% were currently on ART, with 87.4% of these estimated to have suppressed viral load at the time of the survey. These estimates varied by sex, age, and geo-location type. Relatively higher percentages across all three indicators for women compared to men were observed: 88.7% versus 78.2% for those aware of their status, 72.3% versus 67.7% for on ART, and 89.8% versus 82.3% for viral suppression. Knowing one’s positive HIV status increased with age: 74.0, 85.8, and 88.1% for age groups 15–24 years old, 25–49 years old and 50–64 years old, although for those 65 years and older, 78.7% knew their HIV positive status. A similar pattern was observed for the 2nd 90, among those who knew their HIV positive status, 51.7% of 15 to 24 year olds, 70.5% of those aged 25–49 years old, 82.9% of those aged 50–64 years old and 82.4% of those aged 65 years or older were currently on ART. Viral suppression for the above mentioned aged groups, among those who were on ART was 85.2, 87.2, 89.5, and 84.6% respectively. The 90–90–90 indicators for urban areas were 87.7, 66.5, and 87.2%, for rural settings was 85.8, 79.8, and 88.4%, while in commercial farming communities it was 56.2, 67.6 and 81.4%.

**Conclusions:**

South Africa appears to be on track to achieve the first 90 indicator by 2020. However, it is behind on the second 90 indicator with ART coverage that was ~ 20-percentage points below the target among people who knew their HIV status, this indicates deficiencies around linkage to and retention on ART. Overall viral suppression among those on ART is approaching the target at 87.4%, but this must be interpreted in the context of low reported ART coverage as well as with variation by age and sex. Targeted diagnosis, awareness, and treatment programs for men, young people aged 15–24 years old, people who reside in farming communities, and in specific provinces are needed. More nuanced 90–90–90 estimates within provinces, specifically looking at more granular sub-national level (e.g. districts), are needed to identify gaps in specific regions and to inform provincial interventions.

## Background

The 90–90–90 targets are that 90% of people living with HIV (PLHIV) know their HIV status, 90% of people who know their status be on antiretroviral (ARVs), and 90% of all patients receiving antiretroviral therapy (ART) are virally suppressed by 2020 [28]. Modelling exercises showed that achievement of these targets could reduce the number of new infections to levels that may achieve HIV/AIDS epidemic control by 2030 [27].

The HIV epidemic in South Africa, with its estimated 7.1 million PLHIV, accounted for approximately 20% of the 36.9 million PLHIV in the world in 2017 [28]. In that year, the global 90–90–90 indicators were modelled at 75, 79, and 81% for those who knew their HIV status, those on ART who knew their status, and those who had suppressed viral load who were on ART and knew their HIV status, respectively [28]. For South Africa, modelled estimates from the 2015 Thembisa model estimate the indicators at: 85.5, 56.9, and 78.4% [9]. Evaluating the 90–90–90 indicators within South Africa is critical in ensuring progress towards achieving these targets to curb the HIV epidemic nationally and worldwide.

In 2015, the World Health Organisation (WHO) recommended that HIV ARV treatment should start as soon as possible after diagnosis of HIV infection regardless of CD4 count and/or WHO clinical stage [29]. This recommendation, referred to as Universal Test and Treat (UTT), was informed by several studies that showed that early access to ART reduces viral load to very low or undetectable levels, resulting in better health outcomes ([6, 23] and a reduced risk of onward transmission of the virus [21, 26]. South Africa adopted the UTT strategy and rolled out UTT guidelines for the public healthcare sector in September 2016 [17].

Several multi-country studies inclusive of South African sites have evaluated UTT using the 90–90–90 targets. Hayes et al. [7], estimated that 78% of men and 87% of women were aware of their HIV-positive status (i.e. 1st 90) of which 74% of men and 73% of women were on ART (i.e. 2nd 90). Relative to the 90–90–90 cascade measure, the overall percentage of people on ART among all PLHIV was 61% a year after the intervention had started. In the Treatment as Prevention (TasP) trial, 92% of PLHIV indicated that they knew their HIV status, but only 49% were on ART with 93% of these having achieved viral suppression [8]. These studies cover specific geographic areas rather than national in scope.

The Human Sciences Research Council undertook the Fifth South African National HIV Prevalence, Incidence, Behaviour and Communication Survey (SABSSM V) in 2017/8 [25]. This is the fifth in a series of cross-sectional population-based household surveys. Previously such surveys were completed in 2002, 2005, 2008, and 2012. The current survey provides an opportunity to report on the progress of South Africa towards achieving the 90–90–90 targets using data collected from a population-based nationally representative sample following the rollout of UTT nationwide.

## Methods

Details on the SABSSM survey methodology have been described elsewhere [25]. In summary, the South African 2015 national population survey sampling frame was used to randomly select 1000 small area layers (SALs) from a total 103,000 SALs nationwide as defined by Statistics South Africa (STATS-SA, 2017). The sample was further stratified by province and locality type (urban, rural, and farming communities). From each SAL, 15 households or visiting points (VPs) were selected using systematic sampling to identify and enroll individuals into the survey. All members of selected households were invited to participate in the survey. The study excluded facilities and individuals living in education institutions, assisted living homes, hospitals, and those living in uniformed-service barracks. A total of 12,435 VPs from the target of 15,000 (82.9%) VPs were approached, and 11,776 (94.7%) were determined to be valid VPs. A VP was invalid if the selected house was demolished, no one lived in it, or if it was a non-residential structure. Amongst all valid households, 82.2% of household heads gave permission for their households to take part in the survey. Data collection took place from November 2017 to March 2018.

The study received ethical clearance from the Human Sciences Research Council Research Ethics Committee (REC 4/18/11/15) and The US Centers for Disease Control (CDC). In compliance to this ethical clearance, adults, 18 years or older provided written informed consent, while for children, younger than 18 years old, parental/guardian informed written consent and the minor’s assent were obtained before participants took part in the study. Household data for socio-demographic characteristics such as age, sex, race, education, and household assets were collected. Individual level data were also collected on health, sexual behavior, knowledge and perception of HIV and TB. Dried Blood Spots (DBS) were also collected to test for HIV status, recency of infection, exposure to antiretroviral (ARV) drugs, ART resistance, and levels of viral suppression. For this analysis, data for individuals aged 15 years and older were used.

### HIV, ART and viral load testing

Dried Blood Spot (DBS) samples were used for national and sub-national estimates of HIV prevalence, incidence, ARV exposure, HIV drug resistance, and viral suppression. DBS samples were collected by finger prick from consenting individuals. To test for HIV infection, three enzyme immunoassays (EIA) (Roche Elecys HIV Ag/Ab assay, Roche Diagnostics, Mannheim, Germany and Genescreen Ultra HIV Ag/Ab assay, Bio-Rad Laboratories, California, USA) were used. Two assays were used to confirm a result, and in instances where there was a discordant result between the two tests, the third test was used as a tiebreaker. All samples testing HIV positive on two EIAs were subjected to a nucleic acid amplification test (COBAS AmpliPrep/Cobas Taqman HIV-1 Qualitative Test, v2.0, Roche Molecular Systems, New Jersey, USA) for the final interpretation of the test result. High Performance Liquid Chromatography (HPLC) coupled with Tandem Mass Spectrometry was used to test for antiretroviral analytes in HIV-positive specimens.

Viral load testing on confirmed HIV-positive specimens was performed using the recommended testing platform for HIV-1 Ribonucleic acid (RNA) testing in DBS samples (Abbott m2000 HIV Real-Time System, Abbott Molecular Inc., Des Plaines, Illinois, USA).

### Definition of 90–90–90 indicators

The ‘conditional’ 90–90–90 indicator is defined as:

1. First 90/Diagnosed - weighted population percent of individuals who tested HIV positive and reported that they knew their HIV status or had antiretroviral analytes in their blood (numerator) out of all individuals who tested HIV positive (denominator).
2. Second 90/on ART - weighted population percent of those testing positive for antiretroviral analytes (numerator) out of all those who knew their HIV status as defined numerator of 1 above.
3. Third 90/Virally suppressed - weighted population percent of those who had viral loads of less than 1000 copies per ml (numerator) out of all those who had antiretroviral analytes (denominator) as defined numerator of 2 above.

The results are reported by sex, age, self-reported race (black African and other), geographical type (urban, traditional rural areas, or commercial farming communities) and by each of the nine provinces in South Africa. Weighted population percentages of the first 90 vs the second 90, and the second 90 vs the third 90 were plotted by province and shown relative to each of the 90 targets to assess achievement of the targets provincially.

Additionally, gaps to achieving the 90–90–90 targets were reported among all PLHIV to demonstrate the scale of the epidemic and inform programs. The number of individuals nationally who knew their status, those on ART and those with viral suppression among all PLHIV and the numbers needed to achieve the targets were estimated using population weights. Similar to the national presentation of gaps to achievement of the 90–90-90 targets, differences in percentages and numbers of individuals for those who knew their status, those on ART, and those with viral suppression among all PLHIV were estimated for each province.

Population weights based on province, sex, age, and race were used to account for the complex survey design and non-response, and weighted data analysis was implemented using “*svyset*” in Stata. Estimates are reported with 95% confidence intervals (CI) as appropriate. Stata Statistical Software Release 15.0 (College Station, Texas: Stata Corporation, USA) was used to conduct analyses of the data.

## Results

A total of 39,132 participants of all ages from 11,776 households were eligible to participate in the main study. Altogether, 93.6% agreed to be interviewed and, 61.1% agreed to provide specimens for HIV testing. Agreement to be interviewed and to provide a blood sample varied by sex (women - 64.3% vs men – 57.7%), age (15 to 24 years – 66.9%, 25 to 49 years old – 61.5%, 50–64 year olds – 64.4% and 65 years and older – 69.7%), race (Black African – 65.4%, White – 42.3%, Coloured 61.7%, and Asian – 42.6%), and geographic area (urban – 57.3%, rural/traditional – 69.3% and commercial farm areas – 65.1%).

Overall HIV prevalence among people aged 15 years or older was estimated at 18.8% (95% CI 17.5–20.1%), translating to 7,467,321 people infected with HIV in South Africa. The 90–90–90 indicator estimates for South Africa in 2017 were 84.8% (95% CI 81.6–87.6) of PLHIV knew their status, of those, 70.7% (95% CI 67.6–73.7) were on ART, and of those aware of their status and on ART, 87.4% (95% CI 84.9–89.6) had suppressed viral load (Table 1).

**Table 1:** 90–90 – 90 indicators for a national representative sample in South Africa, 2017 among those aged 15 years and above

Variable	1st 90 - Diagnosed & Know HIV-Positive Status			2nd 90 - on ART			3rd 90 - Virally Suppressed		
	%	95% CI	n	%	95% CI	n	%	95% CI	n
**Sex**									
Male	78.2	[72.4–83.1]	808	67.7	[62.2–72.7]	566	82.3	[76.7–86.7]	384
Female	88.7	[85.7–91.1]	2011	72.3	[68.9–75.4]	1681	89.8	[87.0–92.0]	1203
**Age (years)**									
15–24	74.0	[63.6–82.2]	336	51.7	[43.2–60.0]	240	85.2	[77.3–90.6]	131
25–49	85.8	[82.2–88.8]	1959	70.5	[67.0–73.9]	1576	87.2	[84.3–89.7]	1111
50–64	88.1	[81.7–92.5]	461	82.9	[77.1–87.4]	381	89.5	[83.3–93.6]	306
65+	78.7	[63.1–88.9]	63	82.4	[66.3–91.7]	50	84.6	[68.9–93.2]	39
**Sex – Age (years)**									
Male 15–24	68.3	[55.4–79.0]	87	58.8	[41.1–74.5]	50	80.6	[63.2–91.0]	32
Female 15–24	76.4	[62.2–86.5]	249	49.1	[40.5–57.7]	190	87.1	[77.7–92.9]	99
Male 25–49	77.6	[70.6–83.3]	558	64.2	[57.8–70.1]	393	81.6	[74.5–87.0]	249
Female 25–49	90.6	[87.0–93.4]	1401	73.7	[69.9–77.2]	1183	89.7	[86.5–92.2]	862
Male 50–64	85.6	[74.7–92.2]	140	87.5	[77.2–93.5]	103	84.6	[73.4–91.6]	88
Female 50–64	89.8	[83.3–93.9]	321	80.2	[72.8–86.0]	278	92.7	[86.9–96.0]	218
Male 65+	93.9	[72.0–98.9]	23	86.6	[59.1–96.6]	20	91.3	[57.4–98.8]	15
Female 65+	71.6	[51.9–85.5]	40	79.9	[57.4–92.2]	30	80.5	[59.5–92.0]	24
**Race groups**									
Black African	85.3	[82.1–88.0]	2573	70.8	[67.6–73.8]	2087	87.3	[84.6–89.6]	1492
Other	73.1	[54.1–86.2]	246	68.2	[54.1–79.6]	160	92.7	[84.6–96.7]	95
**Geographic type**									
Urban area	87.7	[84.5–90.2]	1505	66.5	[62.6–70.1]	1282	87.2	[83.8–89.9]	874
Rural/Traditional	85.8	[80.3–89.9]	899	79.8	[75.0–84.0]	785	88.4	[83.7–91.9]	602
Commercial Farms	56.2	[37.7–73.1]	415	67.6	[54.9–78.2]	180	81.4	[71.4–88.5]	111
**Province**									
Western Cape	80.9	[71.9–87.6]	223	67.2	[58.0–75.3]	163	89.0	[77.7–95.0]	100
Eastern Cape	89.3	[83.4–93.3]	325	76.9	[65.2–85.5]	282	90.4	[81.7–95.2]	207
Northern Cape	74.3	[60.9–84.2]	182	72.5	[59.0–82.8]	130	87.5	[75.0–94.2]	89
Free State	91.1	[86.0–94.5]	270	68.6	[58.0–77.6]	191	90.0	[81.3–94.9]	131
KwaZulu-Natal	86.3	[79.2–91.2]	637	79.4	[74.4–83.6]	520	85.5	[79.8–89.8]	410
North West	77.9	[59.4–89.5]	295	64.8	[57.9–71.2]	238	90.3	[84.6–94.0]	152
Gauteng	86.5	[80.5–90.9]	324	60.8	[53.3–67.7]	273	87.7	[80.7–92.3]	170
Mpumalanga	84.3	[71.5–92.0]	342	73.2	[65.8–79.6]	284	83.1	[75.4–88.8]	211
Limpopo	76.0	[55.1–89.1]	221	68.7	[58.4–77.4]	166	88.1	[81.4–92.6]	117
**Total**	84.8	[81.6–87.6]	2819	70.7	[67.6–73.7]	2247	87.4	[84.9–89.6]	1587

**Fig. 1:**
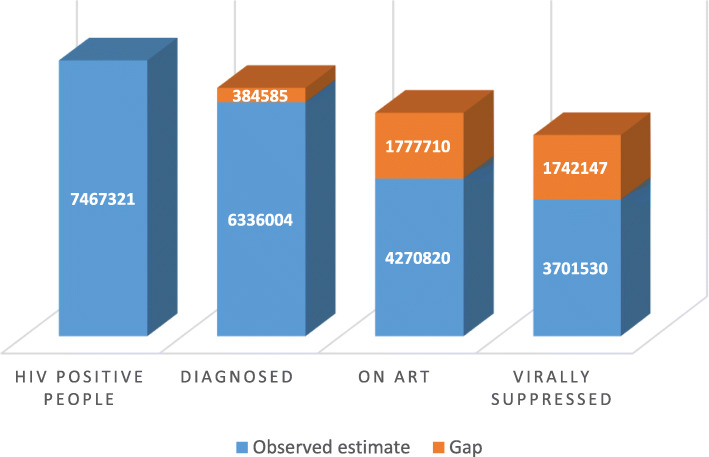
Number of people who know their HIV status, on ART and Virally Suppressed. Numbers of individuals who know their HIV status, on ART and virally suppressed based on the 90–90–90 targets and the gaps to achieve the targets, South Africa 2017

In order to achieve the 90–90–90 targets by 2020, a total of 6,720,589 people living with HIV (PLHIV) aged 15 years and older need to be diagnosed, 6,048,530 initiated on ART, and 5,443,677 of PLHIV on ART must achieve viral suppression (Fig. 1**)**. The gaps in the numbers of people along the treatment cascade in order to achieve these were: an additional 384,585 PLHIV to be diagnosed, an additional 1,777,710 individuals initiated on ART, and an additional 1,742,147 PLHIV to achieving viral suppression (Fig. 1).

**Fig. 2:**
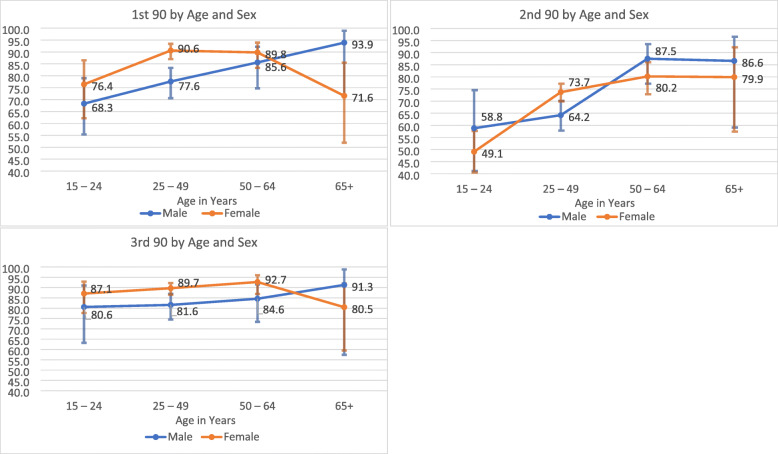
**a**, **b** and **c**: 90–90 – 90 Indicators by Age and Sex. **a**. 1st 90 by Age and Sex - Percentage of PLHIV in each group aware of their HIV status, **b**. 2nd 90 by Age and Sex - Of those who knew their status, those who tested positive for ARVs, **c**. 3rd 90 by Age and Sex - Of those on ART, those who were virally suppressed, South Africa 2017 Fig. 3: Percent of PLHIV diagnosed (1st 90) versus those diagnosed who are on ART (2nd 90), South Africa 2017

Proportionately, more women than men among PLHIV knew their HIV status, 88.7% (95% CI: 85.7–91.1) versus 78.2% (95% CI: 72.4–83.1), respectively. Of those who knew their HIV status and who were on ART, women were more likely than men to be virally suppressed at 89.8% (95% CI, 87.0–92.0) versus 82.3% (95% CI, 76.7–86.7) (Table 1, Fig. 2a-c.).

Point estimates for awareness of HIV status were higher in older age groups: 74.0% (95% CI: 63.6–82.2), 85.8% (95% CI: 82.2–88.8), and 88.1% (95% CI: 81.7–92.5) for 15–24 year olds, 25–49 year olds and 50–64 year olds respectively, with a slight dip, at 78.7% (95% CI: 63.1–88.9) for 65 year olds or older PLHIV. The second 90 target, (i.e. those on ART among those who knew their HIV status) was the lowest, 51.7% (95% CI: 43.2–60.0) for 15–24 year olds compared to the older age groups with the next closest age group, 25–49 year olds at 70.5% (95% CI: 67.0–73.9). For the third 90 estimate, viral suppression was near to the 90% target (range 84.6–89.5%) for all age groups.

Among PLHIV, a higher proportion, 90.6% (95% CI: 87.0–93.4), of women aged 25–49 years old were aware of their HIV status (i.e. 1st 90) compared to their male peers, 77.6% (95% CI: 70.6–83.3), see Fig. 2 a. Differences were not statistically significant for men and women in the other age categories for the first 90 estimates. The trend for the first 90 among men was linear by age category, lowest among 15–24-year olds at 68.3% (95% CI: 55.4–79.0) to 93.9% (95% CI: 72.0–98.9) among men 65 years and older. The trend was an inverted U-shape for women, lowest among 15–24 year olds at 76.4%, (95% CI: 62.2–86.5) and highest among 50–64 years olds 89.8%, (95%CI: 83.3–93.9), but dropping among 65 years and older women at 71.6%, (95% CI: 51.9–85.5).

For the second 90, those aged 15–24 years for men and women, had the lowest proportion of individuals on ART at 58.8% (95% CI: 41.1–74.5) and 49.1% (95% CI: 40.5–57.7), (Fig. 2b) respectively. The highest proportions receiving ART was seen in females aged 25–49 years [73.7% (95% CI: 69.9–77.2)] and in older males aged 50–64 years [87.5% (95% CI: 77.2–93.5)], males 65 years or older 86.6 (95% CI 59.1–96.6) and females 50 to 64 years old, 80.2% (95% CI 72.8–86.0).

Among PLHIV aware of their status and on ART, all sex-age groups’ the third 90 indicators were above 80% with the lowest at 80.5% (95% CI: 74.5–87.0) in women aged 65 years or older and the highest in women aged 50–64 years at 92.7% (95% CI: 86.9–96.0).

The 90–90–90-point estimates for Black Africans were, 85.3% knew their status, 70.8% were on ART and 87.3% were virally suppressed. This is in comparison with the respective estimates of 73.1, 68.2 and 92.7% for the other racial groups combined that include those that self-reported as White, Coloured, or Indian. Commercial farming communities were notable for much lower knowledge of status than the other geographic areas, with a 90–90–90 cascade of 56.2, 67.6, and 81.4% in commercial farming communities compared to cascades of 87.7, 66.5 and 87.2% in urban and 85.8, 79.8 and 88.4% in rural/traditional communities.

**Fig. 3:**
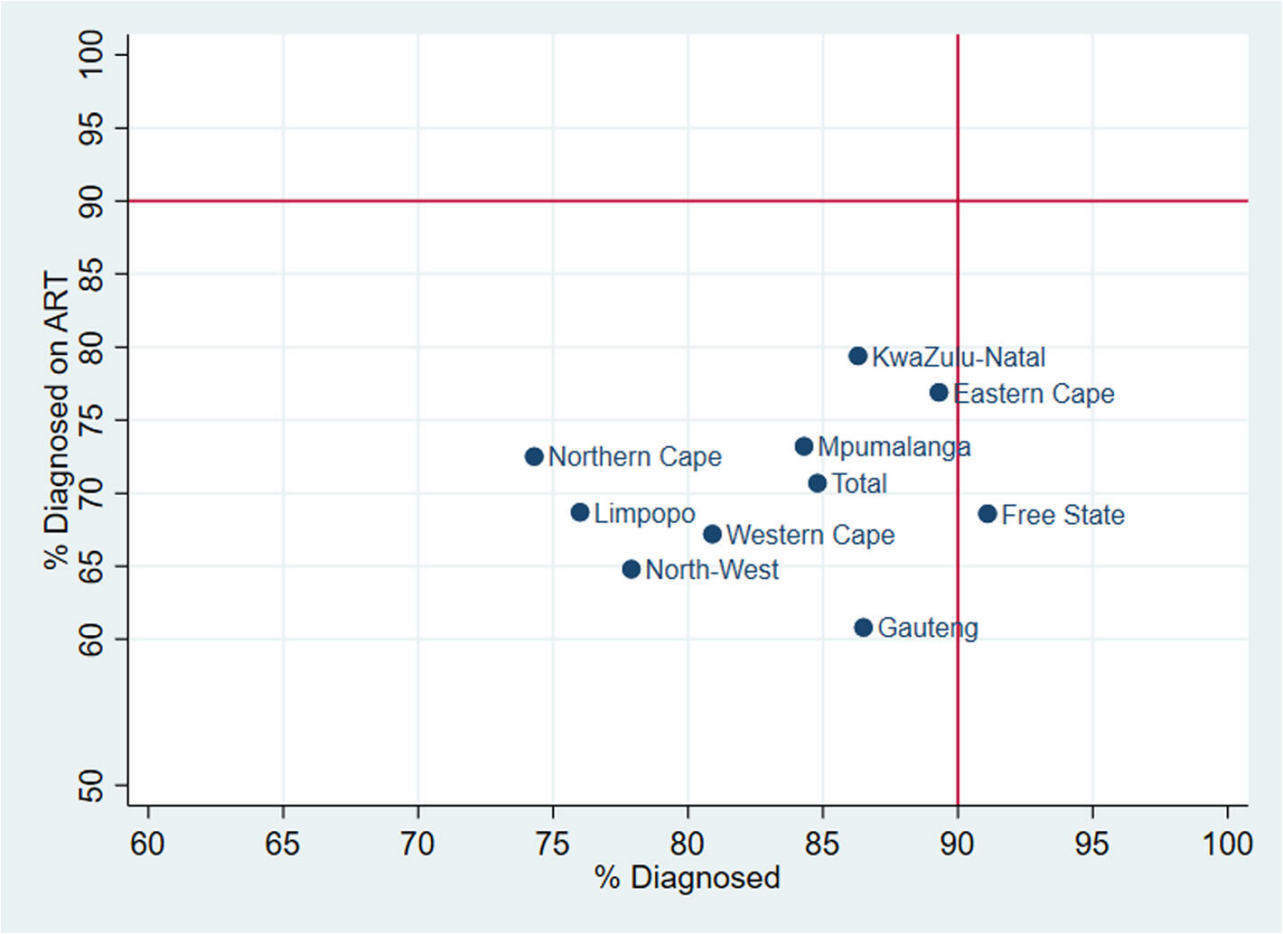
Percent of PLHIV diagnosed (1st 90) versus those diagnosed who are on ART (2nd 90), South Africa 2017

Geographically, the Free State Province (91.1%) had achieved the first 90 target, and the Eastern Cape Province (89.3%) was close to achieving the same target as seen in Table 1. Kwazulu-Natal (86.3%) and Gauteng (86.5%) provinces also had fairly high rates of individuals who knew their status, and the provinces with the lowest percentages of PLHIV aware of their status were Limpopo (76.0%), the North West (77.9%), and the Northern Cape (74.4%) (Table 1 and Fig. 3). ART coverage among those knowledgeable of their status was highest in the provinces of KwaZulu-Natal (79.4%) and the Eastern Cape (76.9%), and it was lowest in the Gauteng (60.8%) and the North West provinces (64.8%).

Figure 3 shows a plot of PLHIV aware of their status versus those PLHIV who know their status and are on ART by province. As no provinces have exceeded the targeted ART coverage rate of 90%, no provinces fell into the top two quadrants. The bottom left quadrant shows inadequate HIV diagnosis rates and ART coverage, while the bottom right quadrant shows provinces that have exceeded the targeted diagnosis rates but not ART coverage.

**Fig. 4:**
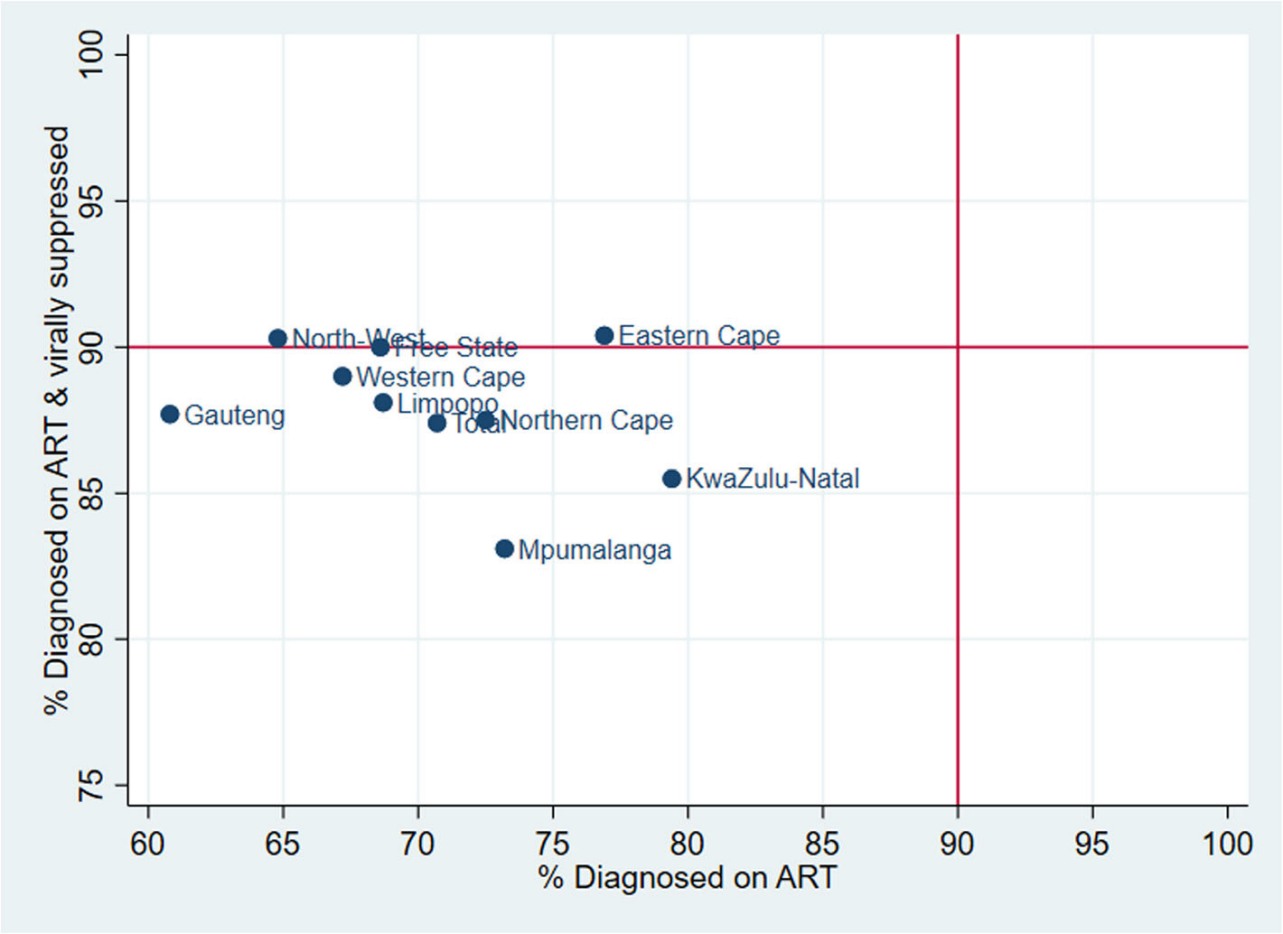
Percent of PLHIV diagnosed who are on ART (2nd 90) versus percentage on ART who are virally suppressed (3rd 90), South Africa 2017

The third 90 target for viral suppression among those knowledgeable of their HIV status and on ART was achieved or close to being achieved in Western Cape (89.0%), Eastern Cape (90.4%), North West (90.3) and the Free State (90.0%) provinces. Only Mpumalanga province (83.1%) had a third-90 indicator value below 85% (Fig. 4**)**.

**Fig. 5:**
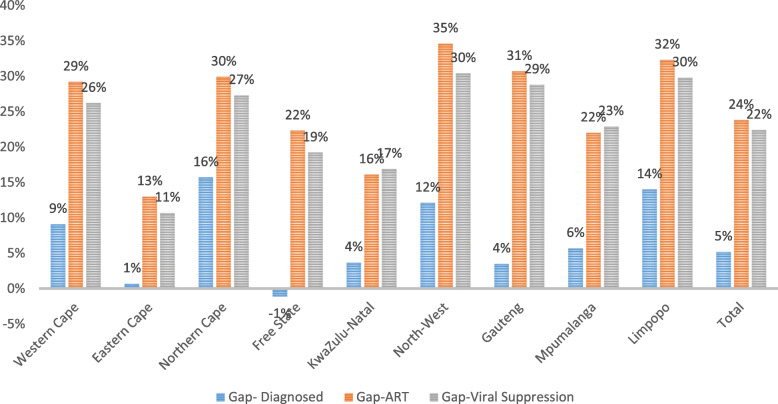
Percentage point gaps in diagnosed (1st 90), on ART, and virally suppressed by Province

The number of people needed (gap) to achieve the 90–90–90 targets, are given in Table 2, while the percentage point gaps for achieving the 90–90–90 targets for each province are shown in Fig. 5. The gap on the percentage of awareness of status among PLHIV ranged from 15.7 percentage points (or 13,872 individuals) in the Northern Cape Province to − 1.1 percentage points (4493 individuals above the target) in the Free State Province. Using 81% (i.e. 1st 90*2nd 90) as the overall ART coverage target among all PLHIV, the largest gaps on ART coverage among PLHIV were observed in the North West Province (34.6 percentage points, 13,1846 individuals), Limpopo Province (32.3 percentage points, 13,2937) and Gauteng Province (30.7 percentage points, 417,184 individuals) while the Eastern Cape Province (13.0 percentage points, 98,016 individuals) and Kwazulu-Natal Province (16.1 percentage points, 23,3581 individuals) had the smallest gaps to achieve the 81% target.

**Table 2:** Gaps in the number of individuals diagnosed (1st 90), on ART and virally suppressed by Province, based on the 90, 81 and 73% cascade^a^

Province	PLHIV	Gap – diagnosed	Gap – on ART	Gap – Viral Suppression
Western Cape	538,472	44,101	114,547	126,972
Eastern Cape	938,221	5911	98,016	90,351
Northern Cape	98,176	13,872	19,619	24,122
Free State	453,821	−4493	82,984	78,420
KwaZulu-Natal	1,868,523	62,222	233,581	284,202
North-West	543,204	59,155	131,846	148,621
Gauteng	1,745,334	54,978	417,184	452,391
Mpumalanga	680,131	34,891	113,534	140,175
Limpopo	601,441	75,782	132,937	161,306
Total	7,467,323	346,419	1,344,248	1,506,560

^a^Displayed gaps in numbers are differences in counts from achieving 90% for diagnosed, 81% (i.e., 0.90 multiplied by 0.90) of all HIV infected people on ART, and 73% (i.e., 0.90 multiplied by 0.90 multiplied by 0.90) of all HIV infected individuals achieved viral suppression

The gap on this second 90 for the cascade has a relational influence on the third 90 or the 73% (i.e. 1st 90*2nd 90 *3^rd^90) target level to be achieved. Thus, the North West Province (30.4 percentage points, 148,621 individuals), Limpopo Province (29.8 percentage points, 161,306 individuals), and Gauteng Province (28.8 percentage points, 452,391 individuals) had the largest gaps to reach the 73% target for viral suppression among all PLHIV, whereas the Eastern Cape Province again had the lowest gap (10 percentage points, 90,351 individuals).

The highest need for diagnosis (1st 90) in terms of absolute numbers was in Limpopo Province (75,782), KwaZulu-Natal Province (62,222), North West Province (59,155), and Gauteng Province (54,978), while the highest needs for ART provision were in Gauteng Province (417,184), KwaZulu-Natal Province (233,581), Limpopo Province (132,937), and North West Province (131,846).

## Discussion

In South Africa 84.8% of PLHIV were aware of their status, of those, 70.7% on ART, and of those knowledgeable of their status and on ART, 87.4% had suppressed viral load in 2017. The country had comparable figures on these 90–90–90 indicators with neighbouring countries for the first and third 90, while the second 90 was relatively lower. In nationally representative Population-Based HIV Impact Assessment (PHIA) surveys carried out in several countries in the Southern Africa region in 2016/2017, the 90–90–90 indicators were: 74, 87, and 87% for diagnosed, on ART and virally suppressed respectively for Zimbabwe, 73, 89, and 91% for Malawi, 85, 87 and 92% for Swaziland, and 81, 92, and 88% for Lesotho [20].^1^ Being on ART was self-reported in all these PHIAs study countries except in Lesotho where self-reporting was used together with laboratory detection of ART analytes among PLHIV.

The results demonstrate a significant gap in the 90–90–90 indicator for South Africa for, ART coverage among diagnosed PLHIV. Although the proportion of PLHIV aware of their status who are on treatment are higher than estimates in previous years, the current rate of increase is not yet sufficient to meet the 90–90–90 targets. Leigh Johnson et al. estimated that 56.9% of diagnosed HIV adults were on ART in 2015 (Johnson, Dorrington et al. 2017). In 2012, 31.2% or 2.0 million PLHIV were on ART [24]. Estimated overall ART coverage from the latest round of this survey increased to 62.3% or 4.4 million among PLHIV by 2017. South Africa adopted UTT into its treatment guidelines in September 2016, shortly before data collection began [17], and the gains from this programmatic shift may not be fully reflected in this survey. Clearly, additional programmatic focus on the second 90, particularly among select populations e.g. adolescents, males will be required if the 90–90–90 targets are to be achieved.

The percentage of PLHIV who knew their status was relatively high at close to 85% with some variation by sex, age and geographic location. This likely includes some individuals who were diagnosed before changes to the UTT guidelines, who at the time of diagnosis were not eligible for ART and have still not been initiated on ART. It is possible that a substantial number of people were not on treatment because they were unaware of the new eligibility criteria or were experiencing other barriers to re-engagement in care. It is important that targeted programmes rapidly identify and facilitate enrolment of these previously diagnosed individuals who were not on treatment.

High proportions of women of reproductive age (25–49 years old) diagnosed and on ART likely reflected increased diagnosis and engagement in care due to pregnancy and Prevention of Mother-to-Child Transmission (PMTCT) programmes. Interestingly, higher proportions of men 50–64 years of age were on ART compared to their younger counterparts. Increased engagement with health facilities due to care-seeking for non-communicable diseases or psychosocial factors are possible explanations for further investigation. Low treatment coverage among young people aged 15–24 years old is of concern. Women in this age group have the highest rates of new HIV infections, (1.5% per year) [25]. The risk of transmission in the immediate seroconversion period is high (Pinkerton, 2007). Thus, it is important for young people to test frequently and if they test HIV-positive, they need to be engaged in care and treatment to reduce the risk of onward transmission. Targeted interventions and changes to HIV programmes may improve diagnosis and ART coverage in this age group. Some of these are scaling up self-testing [14, 19], home-based testing [7, 22], promoting both pre-exposure and post-exposure prophylaxis [2], providing youth and young-people friendly health services [12, 15] and strengthening and streamlining linkage to care and ART initiation [4]. A number of intervention programmes have been designed and implemented targeted especially towards Adolescent Girls and Young Women (AGYW). One such programme, the Determined, Resilient, Empowered, AIDS-free, Mentored and Safe (DREAMS) project was initiated in 2010 in 10 high HIV prevalence countries (PEPFAR, 2017f). The programme was designed based on evidence-based health, educational and social interventions that needed to be implemented urgently, extensively in geographical areas that had the greatest need, thus a broad-based approach with the main aim of reducing HIV incidence among AGYW. The low ART uptake of ART among this group in South Africa, which is one of the DREAMS countries, highlights the need for upscaling HIV Treatment Services, demand creation, testing and counselling, linkage to care for those who are infected, treatment, retention in care and adherence as well as HIV prevention services across the country especially for this group.

There is a need to understand the relatively low levels of ART coverage given the changes in treatment guidelines to UTT in 2016. Previous studies found that being diagnosed with HIV does not necessarily translate to universal uptake of ART even though one is offered treatment [10, 11, 16]. It is not clear what the scale of treatment refusal among PLHIV is. In a study conducted in Soweto, Johannesburg, 20% of newly HIV diagnosed women with low CD4 cell counts and/or who presented with TB refused to be initiated on ART [11]. Some of the reasons given for refusing ART initiation included “feeling healthy”, “unable to disclose to family members”, and “(concern about) side effects”. A study done in the KwaZulu-Natal Province showed that linkage to care was low for newly diagnosed individuals, students in education institutions, and those staying far away from health care facilities [13]. However, from the same study, those who reported positive experiences of ART among their close relatives and friends were more likely to access HIV services.

Fox at el reported retention in care of 63.3% after 6 years of treatment at the national level [3]. They also reported high rates of patients moving between clinics and migration, the so called ‘silent transfer’, thus usually resulting in under-estimation of retention rates from clinics perspectives. The current survey used the presence of ART analytes to measure exposure to ART which in a way measures some indication of adherence. In the 2017/18 financial year the National Department of Health partnered with all provincial departments of health in the country on developing plans to improve the 90–90–90 outcomes, and this resulted in 13 million individuals being tested for HIV and 4.1 million individuals being retained on treatment [18].

There are noticeable variations in the first two 90s, diagnoses and ART coverage between provinces. For example, the Eastern Cape and Free State provinces achieved the first 90 (diagnosis), but they had low values on the second 90 indicator, while the Northern Cape and the North West provinces had low levels on both the first and second 90 indicators. The same can be said when one compares the first two 90s for geographical areas, commercial farms had very low rates of diagnosis and ART coverage compared to the other two geographic areas. Geographical differences in health systems such as waiting times for patients, drug availability, staff and system capacity to diagnose and treat more patients need to be better understood.

It is encouraging to see that for those individuals who were on treatment as measured by bioavailability anti-retroviral analytes in their blood, viral suppression was high and close to the 90% target. However, as the gap analyses demonstrated, turning the HIV epidemic tide through controlled viral suppression can only be achieved if there are sufficiently large numbers of PLHIV who are diagnosed who are also enrolled onto the ART programme.

Unlike other studies where self-reporting is used, the strength of our study is that the results presented in this study are calculated using population-based laboratory HIV, ART and viral load testing. The estimates for ART coverage may be lower than other studies and reflects “on ART”. Individuals on “treatment holidays” or defaulting for more than 14–28 days, the half-life for most ART drugs [1], would have tested ARV negative. Furthermore, our household-based survey captures individuals accessing care who may be missed in existing surveillance systems such as those accessing services in the private healthcare sector.

There are several limitations associated with this study. Ninety four percent (94%) of survey participants agreed to answer the questionnaire, and of these, 61% agreed to provide blood samples for HIV testing, ART testing and viral suppression testing. If PLHIV who provided blood samples are different from PLHIV who were not tested, then the presented results may be biased. Extensive training of enumerators and undertaking multiple visits for those not at home, up to three visits were done to increase participation and minimise such biases.

## Conclusion

In conclusion, South Africa has made enormous strides to meet the 90–90–90 targets over the past 15 years, but it still has a significant way to go to reach them especially among some sub-population groups such as adolescents and young women.

Critical in achieving 90–90–90 targets is the routine monitoring and analysis of the HIV treatment cascade framework that tracks the delivery of services from HIV diagnosis, linkage to care, initiation of ART, adherence and retention in care, and viral suppression [5]. Targeted interventions tailor-made for specific provinces and geographic areas are also needed. To provide more useful results and recommendations that are implementable and can be tracked, it is important to collect district and sub-district level data so that targeted interventions can be implemented at these levels. Those large numbers of individuals who have defaulted on treatment as well as those who have been tested before but were never initiated on treatment should be quickly identified and brought into treatment and care programmes via community-based or other innovative e.g. social media, interventions. Other factors such as substance use, poor health status, mental health and other social/welfare factors (Bulsara, 2018) needs to be addressed if good retention in care and adherence to treatment is to be achieved. If these individuals could return to care, more people could be initiated on ART, and if current high levels of viral suppression among those on ART are maintained, then epidemic control can be achieved.

## Data Availability

The datasets used and/or analysed during the current study are available from the corresponding author on reasonable request.

## Abbreviations

ART: Antiretroviral Therapy
AGYW: Adolescent Girls and Young Women
CDC: Centers for Disease Control
CI: Confidence Interval
DBS: Dried Blood Spot
DREAMS: Determined, Resilient, Empowered, AIDS-free, Mentored and Safe
EIA: Enzyme Immunoassays
HPLC: High Performance Liquid Chromatography
NDoH: National department of Health
PHIA: Population-Based HIV Impact Assessment
PLHIV: People Living with HIV
REC: Research Ethics Committee
PMTCT: Prevention of Mother-to-Child Transmission
RNA: Ribonucleic acid
SABSSM V: Fifth South African National HIV Prevalence, Incidence, Behaviour and Communication survey
SAL: Small Area Layers
STATS SA: Statistics South Africa
TasP: Treatment as Prevention
UNAIDS: Joint United Nations Programme on HIV/AIDS
UTT: Universal Test and Treat
VP: Visiting points
WHO: World Health Organisation

## Footnotes

1 In Zimbabwe and Malawi, the 90–90–90 targets were reported for individuals aged 15 to 64 years old, 15 years and older in Swaziland, similar to this study; and for Lesotho the estimates for those aged 15 to 59 years old were reported.
